# Molecular cloning and functional characterisation of an H^+^-pyrophosphatase from *Iris lactea*

**DOI:** 10.1038/s41598-017-18032-3

**Published:** 2017-12-19

**Authors:** Lin Meng, Shanshan Li, Jingya Guo, Qiang Guo, Peichun Mao, Xiaoxia Tian

**Affiliations:** Beijing Research and Development Center for Grass and Environment, Beijing Academy of Agriculture and Forestry Sciences, Beijing, 100097 P. R. China

## Abstract

Tonoplast H^+^-pyrophosphatases (VPs) mediate vacuolar Na^+^ sequestration, a process important for salt tolerance of plants. The function of VP in the highly drought- and salt-tolerant perennial *Iris lactea* under salt stress is unclear. Here, we isolated *IlVP* from *I*. *lactea* and investigated its function in transgenic tobacco. IlVP was found to comprise 771 amino acid residues and showed 88% similarity with *Arabidopsis* AtVP1. *IlVP* was mainly expressed in shoots and was up-regulated by salt stress. Overexpression of *IlVP* enhanced growth of transgenic tobacco plants compared with wild-type (WT) plants exposed to salt stress. Transgenic plants accumulated higher quantities of Na^+^ and K^+^ in leaves, stems, and roots under salt stress, which caused higher leaf relative water content and decreased cell membrane damage compared with WT plants. Overall, *IlVP* encoding a tonoplast H^+^-pyrophosphatase can reduce Na^+^ toxicity in plant cells through increased sequestration of ions into vacuoles by enhanced H^+^-pyrophosphatase activity.

## Introduction

Plant physiological drought leads to ionic imbalance in cells, depressed functioning of cell membranes and metabolic activity, and even cell death owing to excessive soil Na^+^ concentrations^1^. To cope with salinity stress, strategies adopted by plants cells to Na^+^ compartmentalisation into vacuoles alleviated the cellular Na^+^ toxicity to maintain osmotic balance using Na^+^ as a osmoregulation substance, thus to improve salt tolerance of plant^2^. Previous studies suggested that tonoplast Na^+^/H^+^ antiporters (NHXs) could mediate Na^+^ compartmentation into vacuolar^3^. The process is driven by electrochemical gradient of protons across tonoplast generated by the H^+^- ATPase and H^+^-pyrophosphatase (H^+^-PPase) in tonoplast^4,5^. It has been suggested that H^+^-PPase plays an important role in salt tolerance via the establishment of a transmembrane electrochemical gradient^6,7^. First cloned from *Arabidopsis thaliana*, H^+^-PPase genes have subsequently been cloned from other plants, such as *Hordeum vulgare* ^8^, *Beta valgaris* ^9^, *Pyrus serotina* ^10^, *Triticum aestivum* ^11^, *Thellungiella halophila* ^12^, and *Haloxylon ammodendron* ^13^. H^+^-PPase activity and transcript levels can vary among different plant species, organ types, growth stages, and Na^+^ concentrations in nutrient solutions^14^. In *Daucus carota* ^15^, *Helianthus annuus* ^16^, *Suaeda salsa* ^17^, and *Thellungiella halophila* ^18^ subjected to different NaCl concentrations, tonoplast H^+^-PPase activity was higher in treated plants than in the control, further demonstrating that NaCl may induce an increase in H^+^-PPase activity. In contrast, however, Matsumoto and Chung^19^ reported that H^+^-PPase activity in *Hordeum vulgare* roots treated with 200 mM NaCl was half that of the control, and similar results were obtained in a study of *Mesembryanthemum crystallinum* treated with 400 mM NaCl^20^. These suggest that overexpressing the H^+^-PPase resulted in enhanced resistance to salt in various transgenic plants linked with the increased Na^+^ compartmentation into the vacuoles.

*Iris lactea* Pall. var. *chinensis* (Fisch.) Koidz., a wild perennial monocotyledonous halophyte, is widely distributed in desert steppe and saline lowland meadows in northern China, Siberian regions, eastern Russia, and Mongolia^21^. Moreover, this species has attractive leaves and flowers, a wide range abundant seeds, stronger salt and drought tolerance, higher pest and disease resistance, and easy cultivation, which has become a popular groundcover plant for landscape design and park greenspace construction in northern China because of its ornamental foliage and flower^22,23^. Our previous research showed that the salt sensitive BJCY-ML035 in meadow grassland (37°31′12′′ N, 112°19′00′′ E; altitude 760 m) and the salt tolerant BJCY-ML007 in saline lowland meadow (43°45′15′′ N, 83°10′30′′ E; altitude 1,071 m) were screeed out from the sixteen accessions of *I*. *lactea* in northern China by the comprehensive assessment of salinity soils^24^. Further research suggested that the specific locus ISSR841-220 associated with the VP gene was found in the BJCY-ML007 compared with BJCY-ML035^25^. However, the role of IlVP in the salt tolerance of *I*. *lactea* is still unclear.

To test whether the overexpression of *IlVP* confers improved salt tolerance in plant, we introduced the gene into tobacco to measure and analyse the growth performance and Na^+^, K^+^ concentrations in the transgenic tobacco plants and in wild-type (WT) plants subjected to salinity stress. The results indicate that *IlVP*-mediated compartmentalisation of Na^+^ into vacuoles may play a key role in salt tolerance of plant. This would provide a potential benefits for generating engineered plants to increased tolerance to salinity conditions.

## Results

### Isolation and characterisation of *IlVP*

An 893-bp fragment was first obtained by RT-PCR using degenerate primers P1 and P2 (Supplementary Figure 1). A nucleotide BLAST search revealed that the isolated cDNA fragment shared high sequence homology with many known *VPs* from other plants (e.g. *Oryza sativa*), indicating that a partial potential *VP* had been isolated from *I*. *lactea*. Sequences of the 5′ and 3′ ends were obtained by rapid amplification of cDNA ends (RACE), which yielded products of 1,117 bp and 881 bp, respectively. The open reading frame (ORF) of *IlVP* was 2,316 bp long and encoded a polypeptide protein consisting of 771 amino acid residues (Supplementary Figure 1). The predicted protein had an isoelectric point of 5.16 and a molecular weight of 80.7 kDa. The cDNA sequence of *IlVP* was submitted to GenBank under accession number KY406740.

**Figure 1:**
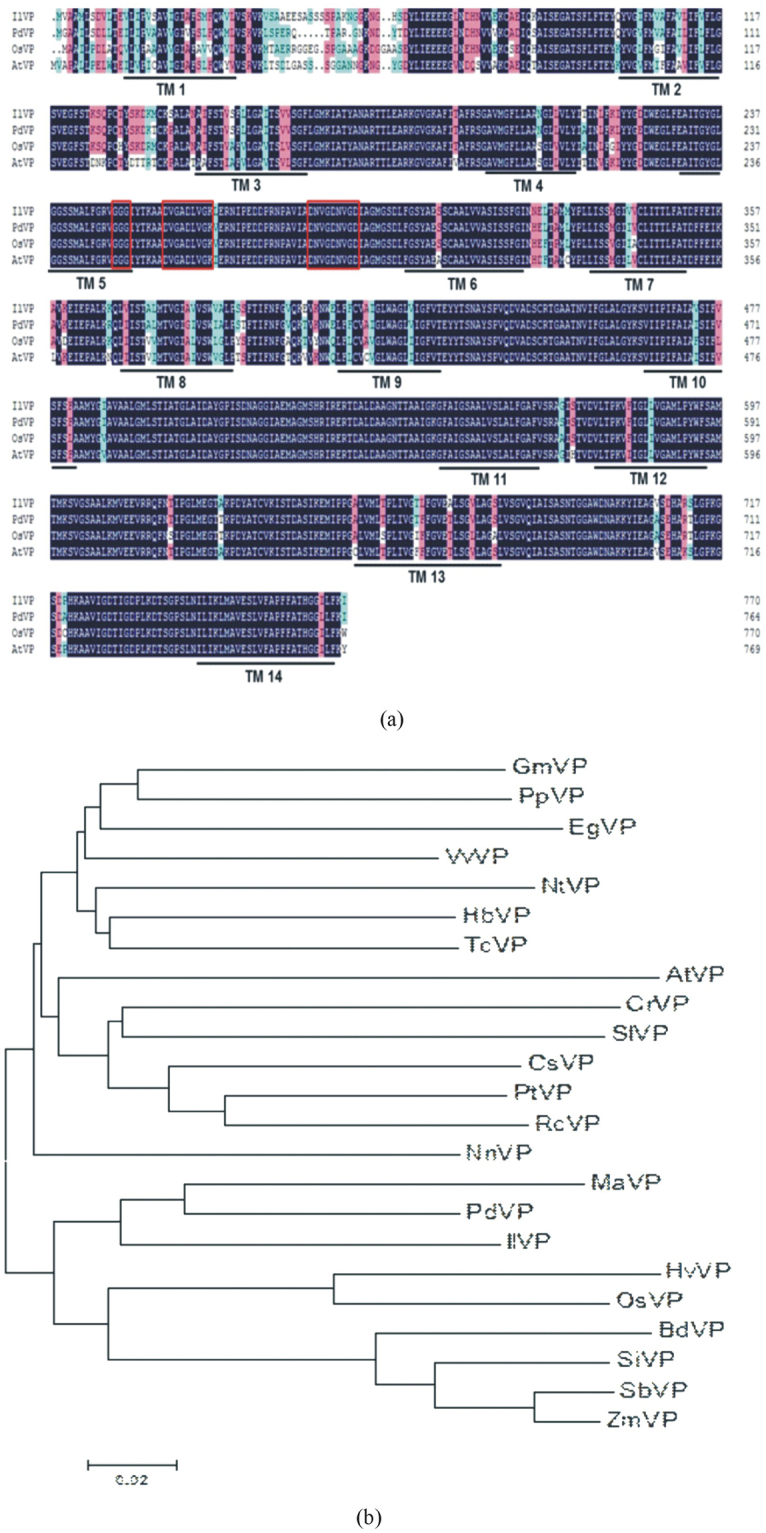
(**a**) Alignment of amino acid sequences of H^+^-PPase genes from *Iris lactea* var. *chinensis* (IlVP) with those from *Phoenix dactylifera* (PdVP), *Oryza sativa* (OsVP), and *Arabidopsis thaliana* (AtVP). Amino acid sequences enclosed in red frames represent the PPi binding sites and activity domains of H^+^-PPase. (**b**) Phylogenetic tree of H^+^-PPase genes from *Iris lactea* and other plant species. Genes and GenBank accession numbers are as follows: *AtVP* (*Arabidopsis thaliana*, NM_101437), *BdVP* (*Brachypodium distachyon*, XM_003564169), *CrVP* (*Chenopodium rubrum*, AF533336), *CsVP* (*Citrus sinensis*, XM_006474322), *EgVP* (*Eucalyptus grandis*, XM_010035677), *GmVP* (*Glycine max*, XM_003528254), *HbVP* (*Hevea brasiliensis*, AY514019), *HvVP* (*Hordeum vulgare*, AK360389), *IlVP* (*Iris lactea*, KY406740), *MaVP* (*Musa acuminata*, XM_009386846), *NtVP* (*Nicotiana tomentosiformis*, XM_009630002), *NnVP* (*Nelumbo nucifera*, XM_010246610), *OsVP* (*Oryza sativa*, D45383), *PdVP* (*Phoenix dactylifera*, XM_008790581), *PpVP* (*Prunus persica*, AF367446), *PtVP* (*Populus trichocarpa*, XM_006381029), *RcVP* (*Ricinus communis*, XM_002530709), *SbVP* (*Sorghum bicolor*, HM143921), *SiVP* (*Setaria italic*, XM_004964638), *SlVP* (*Solanum lycopersicum*, NM_001278976), *TcVP* (*Theobroma cacao*, XM_007023235), *VvVP* (*Vitis vinifera*, XM_002273171), and *ZmVP* (*Zea mays*, BT086232).

Analysis using the TMpred tool indicated that *IlVP* contained 14 transmembrane regions (Fig. 1a). Both of N- and C-terminus were located in vacuole. Multiple sequence alignment showed that the PPi binding site sequences were GGG, DVGADLVGK, and DNVGDNVGD, all located in the loop sequence connecting domains TM5 and TM6 in the cytoplasm. The core VP sequence, essential for implementation of proton transfer functions, was highly conserved and corresponded to that of *PdVP*, *OsVP1*, and *AtVP*. Alignment of H^+^-PPase amino acid sequences of *I*. *lactea* and other plant species showed that *IlVP* was 96%, 93%, and 88% similar to *PdVP*, *OsVP*, and *AtVP*, respectively (Fig. 1b). Phylogenetic analysis indicated that *IlVP* was most closely related to *PdVP* and *MaVP*, and only distantly related to *GmVP* (Fig. 1b). Consequently, *IlVP* may have the same function as other plant vacuolar membrane H^+^-PPases such as *AtVP* and may play an important role in drought resistance and salt tolerance.

### Expression pattern analysis of *IlVP*

**Figure 2:**
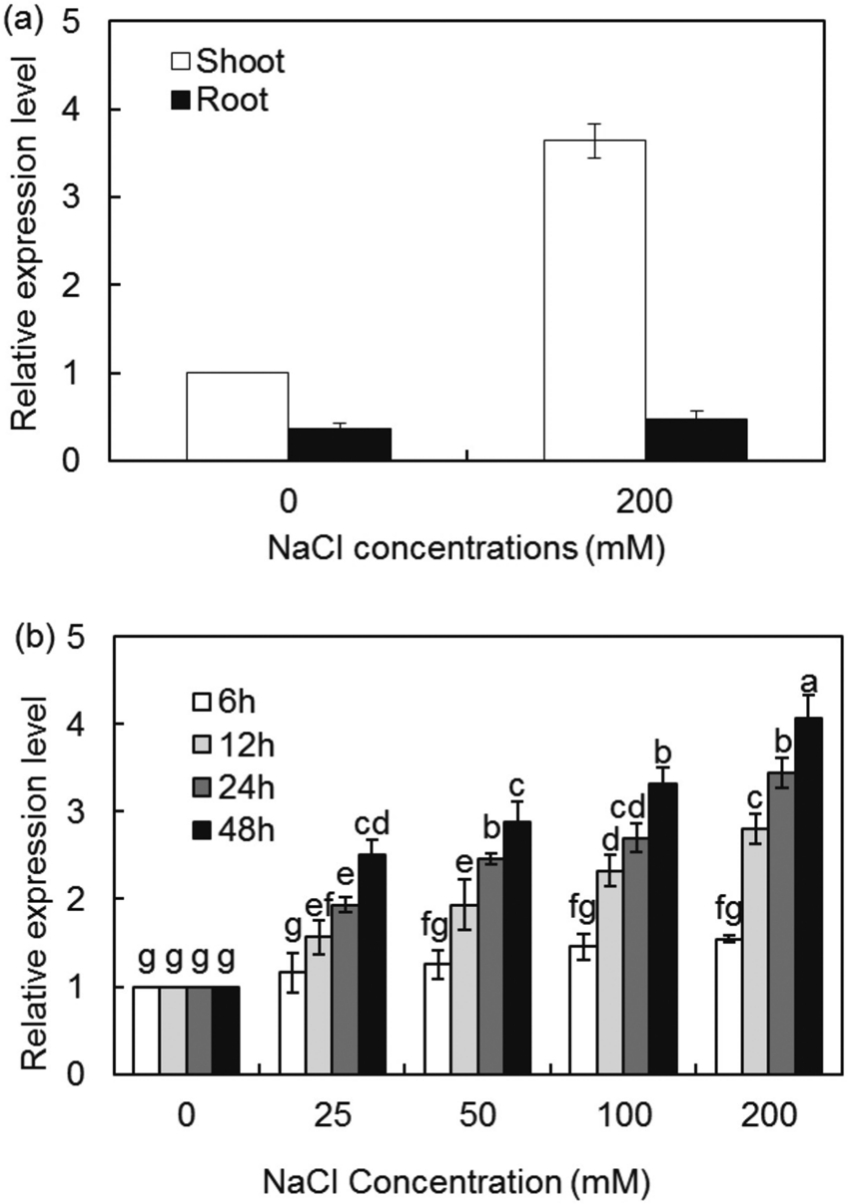
Expression analysis of *IlVP* in shoots and roots of *Iris lactea* var. *chinensis* under different NaCl treatments. The expression levels of *IlVP* in shoot and root under control (0 mM) and 200 mM were normalized with that in shoot in control. (**a**) *IlVP* expression in roots and shoots under control and 200 mM NaCl treatment for 24 h as indicated by quantitative real-time PCR (qRT-PCR); (**b**) *IlVP* expression in shoots after treatment with different concentrations of NaCl (0, 25, 50, 100, and 200 mM) for 0, 6, 12, 24, and 48 h as indicated by qRT-PCR. Each bar represents the mean (*n* = 3), and bars indicate the standard deviation (SD).

To investigate the tissue-specific expression of *IlVP*, plants were subjected to 200 mM NaCl for 24 h. In the absence of NaCl, *IlVP* was constitutively expressed in roots and shoots (Fig. 2a). In the presence of 200 mM NaCl, *IlVP* transcripts were detected in both organs, with level 7.6 times higher in shoots than in roots (Fig. 2a). Subsequently, *I*. *lactea* plants were treated with 0, 25, 50, 100, or 200 mM NaCl for 0, 6, 12, 24, and 48 h. *IlVP* expression levels in shoots increased significantly as salt concentration and stress duration were increased (Fig. 2b). These findings suggest that *IlVP* expression is induced by salt. Compartmentalisation of cytoplasmic Na^+^ into the vacuole would help reduce salt-induced cell damage.

### Production and molecular characterisation of tobacco plants over-expressing *IlVP*

To investigate the potential benefit of transferring *IlVP* into other plant species, we identified eight independent *IlVP* transgenic tobacco lines by PCR amplification (data not shown). To further examine *IlVP* transcript levels in transgenic tobacco, we used Northern hybridisation to analyse *IlVP* expression in young leaves of all PCR-positive lines and WT plants. We observed that the relatively lower expression levels in L4 and the highest expression levels in L18, but had not detected in the WT (Supplementary Figure 2a). We therefore used lines 4 and 18 in the following assay.

Subsequently, we randomly selected four transgenic tobacco lines and determined whether *IlVP* was introduced into the tobacco genome using Southern hybridisation analysis. As shown in Supplementary Figure 2b, only one band was visible in transgenic tobacco lines 4, 6, 14, and 18 after the hybridisation, whereas line 10 yielded two copies (Supplementary Figure 2b). The Southern blot analysis thus confirmed the integration and expression of *IlVP* in tobacco.

In addition, membrane proteins of isolation increased markedly the IlVP protein in T4 and T18 by Western blot analysis compared to WT, and the protein level in T18 was higher than in T4 (Supplementary Figure 2c), expecting that heterologous expression of *IlVP* could enhance salt tolerance in transgenic tobacco.

### Effect of NaCl stress on transgenic tobacco growth

**Figure 3:**
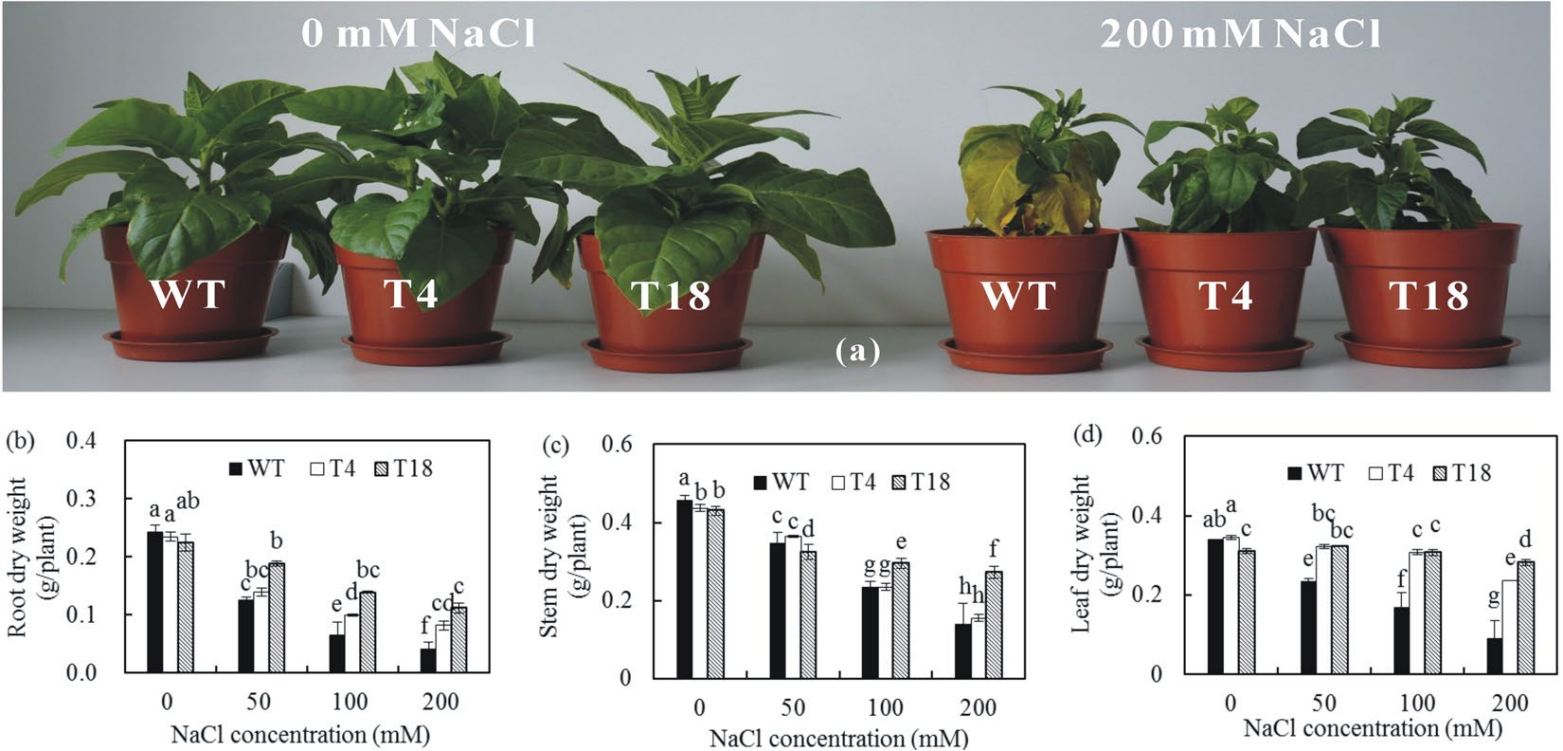
(**a**) Growth of wild-type and *IlVP-*transgenic tobacco plants in response to 200 mM NaCl treatment for 7 days. WT: wild type; T4, T18: transgenic tobacco. (**b**–**d**) Root, stem and leaf dry weight of wild-type and *IlVP-*transgenic tobacco plants in response to salt stress, respectively. Each bar represents the mean (*n* = 7), and error bars indicate the standard deviation (SD). Columns with different letters indicate a significant difference at *P* < 0.05 (Duncan’s multiple range test).

The dry weights of both WT and transgenic tobacco plants decreased gradually with increasing NaCl concentration. The weights of the transgenic tobacco plants declined more slowly, and the dry weights were higher in the transgenic tobacco plants than the WT plants (Fig. 3a). In particular, the dry weights of the roots, stems, and leaves were 2.0-, 1.1-, and 2.6-fold higher in T4 and 2.7-, 1.9-, and 3.1-fold higher in T18, respectively, under 200 mM NaCl compared with the WT plants (Fig. 3b,c,d). The growth of the transgenic plants was thus significantly better under salt stress compared with the WT plants, and this growth increased as the relative expression of *IlVP* increased.

### Effect of NaCl stress on leaf relative water content and plasmalemma permeability

**Figure 4:**
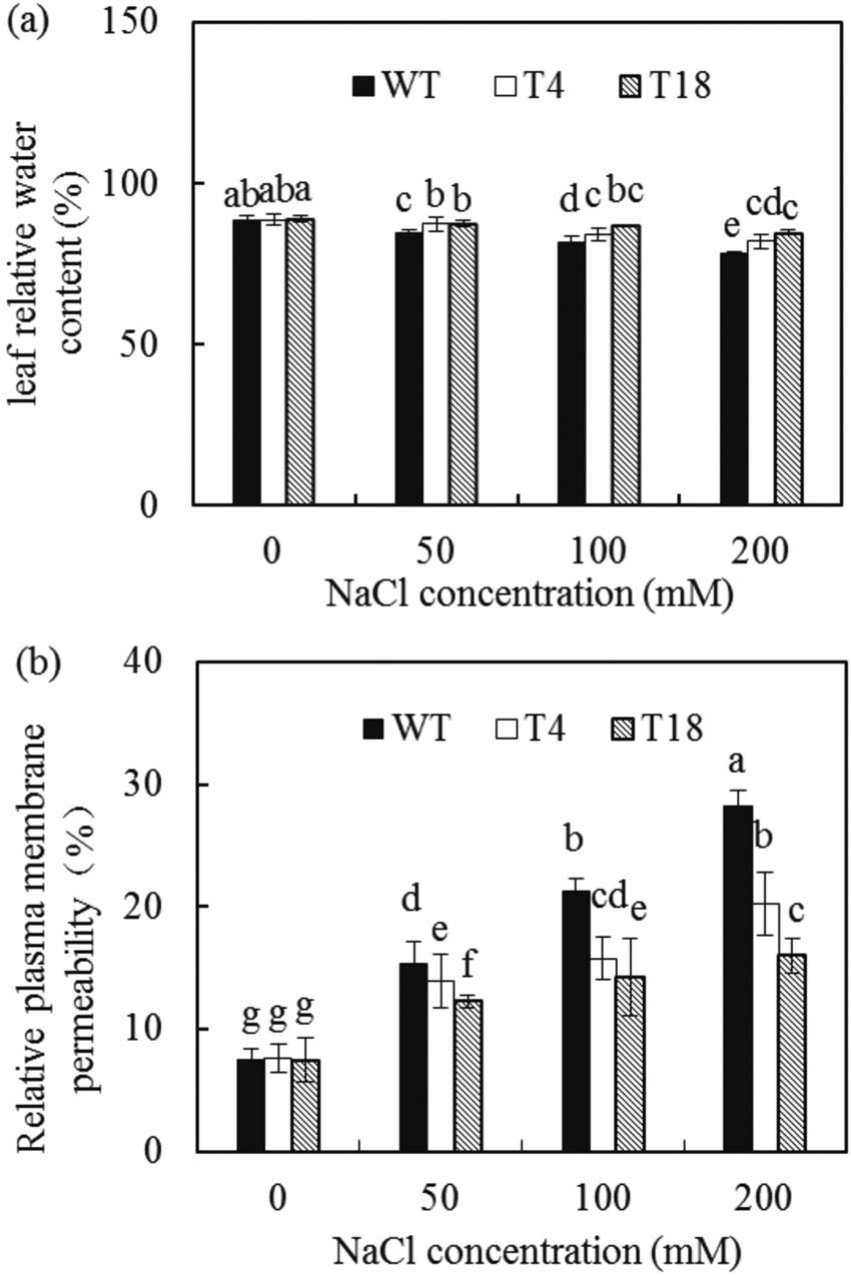
Leaf relative water content (**a**) and relative membrane permeability (**b**) of wild-type and *IlVP-*transgenic tobacco plants in response to salt stress for 7 days. Each bar represents the mean (*n* = 7), and error bars indicate the standard deviation (SD). Columns with different letters indicate a significant difference at *P* < 0.05 (Duncan’s multiple range test).

The relative water content is an important physiological indicator of plant growth status. The plant water content can be divided into free and bound water, with the majority present as free water. The free water ratio can reflect plant salt resistance in response to the metabolic situation. With increasing salt concentration, the leaf relative water content distinctly decreased in both the WT and transgenic tobacco plants compared with the control, but that of the transgenic tobacco plants declined more slowly and remained higher than that of the WT plants (Fig. 4). In response to treatment with 200 mM NaCl, the leaf relative water content of the transgenic tobacco plants was 1.05 times higher in T4 and 1.08 times higher in T18 compared with the WT (Fig. 4a). *IlVP* was therefore found to enhance the salt resistance of the tobacco plants. The water-retention capacity of the transgenic tobacco plants increased significantly under salt stress.

Maintenance of the cell microenvironment and normal metabolism relies on plant cell membranes. The relative plasma membrane permeability of the WT and transgenic tobacco plants increased with NaCl treatment, with a lower increase observed in the transgenic than the WT plants (Fig. 4b). For instance, the relative plasma membrane permeability was 28.3% (T4) and 43.2% (T18) lower than that of the WT plants subjected to 200 mM NaCl treatment for 7 days. Damage to the cell membranes of the transgenic plants under salt stress was thus less severe than that of the WT plants, while the salt resistance of the transgenic plants was higher than that of the WT.

### Effect of NaCl stress on Na^+^ and K^+^ concentrations

**Figure 5:**
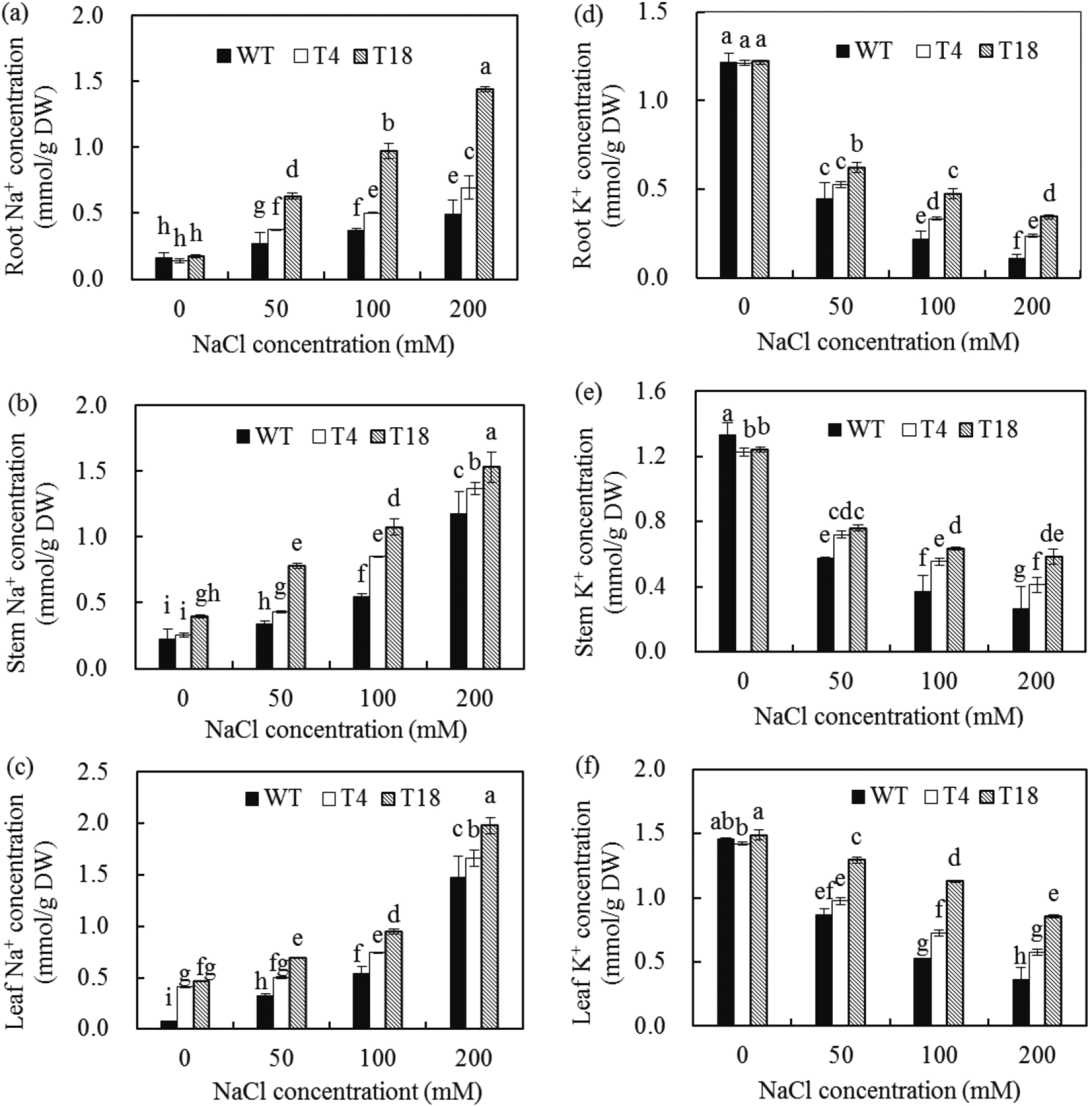
Cation concentration in tissues of wild-type and *IlVP-*transgenic tobacco plants in response to salt stress. Na^+^ (**a**–**c**) and K^+^ (**d**–**f**) concentrations were measured after treatment for 7 days with different NaCl concentrations (0, 50, 100, and 200 mM). Each bar represents the mean (*n* = 7), and error bars indicate the standard deviation (SD). Columns with different letters indicate a significant difference at *P* < 0.05 (Duncan’s multiple range test).

Concentrations of Na^+^ in the tissues (roots, stems, and leaves) of the WT and transgenic plants (T4 and T18) increased with increasing NaCl concentrations; considerably higher increases were observed in the transgenic compared with the WT plants. Under 200 mM NaCl treatment for 7 days, the Na^+^ concentrations in the roots, stems, and leaves of T4 were 38.7%, 15.7% and 12.2% higher, and those of T18 were 188.0%, 29.5% and 33.5% higher, respectively, compared with the WT (Fig. 5a,b,c). The K^+^ concentration in the tissues of transgenic plants was significantly higher than that in the WT plants in the presence of 200 mM NaCl. Although the accumulation of K^+^ in T4, T18 and WT decreased with external NaCl treatment, the tissues of transgenic plants retained more K^+^ (Fig. 5d,e,f). Under NaCl treatment, the transgenic lines showed significantly higher concentrations of Na^+^ and K^+^ than the WT plants.

## Discussion

In a previous study, we determined that *I*. *lactea* has strong salt resistance^24^, and a rapid tissue culture propagation system was subsequently established^26^. It is well known that tonoplast H^+^-PPase is involved in the sequestration of Na^+^ into vacuoles, which contributes to salt tolerance of plants^27^.

The tonoplast H^+^-PPase is encoded by a highly hydrophobic, single-subunit protein with a calculated molecular mass of 80 kDa^28^. In higher plants, H^+^-PPase cDNA commonly contains a 2,283–2,319 bp ORF encoding 761–773 amino acid residues with a deduced calculated molecular mass of 79–81 kDa^29–31^. Our results showed that *IlVP* consists of 2,316 bp, encodes a protein of 771 amino acids with a calculated molecular mass of 80.7 kDa, and contains 14 trans-membrane domains. Two contrasting responses in tonoplast H^+^-PPase activity under salt stress have been reported. Some researchers have reported that H^+^-PPase activity declines in response to salt treatment^19^, whereas other studies have shown that NaCl may enhance H^+^-PPase activity^15,16,18^. H^+^-PPase hydrolytic activity in barley roots and leaves has been found to increase under different salt concentrations^17^. *IlVP* was mainly expressed in shoots under the NaCl concentrations in our study, and transcript abundance of *IlVP* in shoots increased gradually with increasing NaCl concentrations (50 and 200 mM). This response is conducive for compartmentalisation of Na^+^ in the vacuole of the leaf cytoplasm, with a consequent reduction in the salt damage caused to plants^32^. Overexpression of the H^+^-PPase gene may enhance trans-membrane electrochemical gradients and improve secondary transport carrier efficiency across the vacuole membrane under salt stress^33^. A variety of inorganic ions accumulate in the vacuoles to maintain the balance between ionic equilibrium, osmotic equilibrium, and cell turgor-pressure stability; in this way, damage to cells by inorganic ions is reduced, and salt or osmotic stress tolerance of cells is enhanced^14^. Thus, H^+^-PPase plays an important role as a proton pump in the process of salt or osmotic stress resistance and adaptation.

Plant salt resistance is enhanced by excessive expression of tonoplast H^+^-PPase. Overexpression of *SaVP1* in *Arabidopsis* enhances tolerance to drought and salt stresses; this overexpression also results in the up-regulation of several K^+^ and Ca^2+^ channel/transporter genes that show a function similar to that of vacuolar H^+^-PPase from other plants^33^. Overexpression of *KfVP1* increases salt and drought tolerance of *Arabidopsis* ^31^. The *AVP1* protein content in *AVP1*-transgenic *Arabidopsis* seedlings was significantly higher than that of WT plants^34^. Recovery of salt resistance can be achieved by overexpression of *Arabidopsis AVP1* in yeast salt-sensitive *enal* mutants^35^. In one study, inorganic ion accumulation was higher in the roots and leaves of *AVP1* genetically modified alfalfa (*Medicago sativa*) than in WT plants, and the leaf osmotic potential of transgenic plants was reduced. In addition, the salt and drought resistance of the transgenic plants was significantly enhanced^36^. *PvVP1* has been transferred to non-halophytic grass, thus providing a feasible basis to improve the salt resistance of *Paspalum vaginatum* ^37^. Overexpression of the H^+^-PPase gene provides a stimulus for ion compartmentalisation, thereby maintaining the balance between ionic balance and osmotic equilibrium within the cell and enhancing plant salt resistance^10^. In the present study, using the anti-AtVP found that exogenous IlVP protein was largely expressed in transgenic plants by Western blots analysis compared to WT. This suggested the salt tolerance of the *IlVP* transgenic tobacco plants was higher than that of the WT plants. Compared with the WT plants, the physiological indices of the transgenic tobacco plants showed greater stability and slower changes, which indicated that the physiological system of the transgenic tobacco plants had not been severely damaged under NaCl stress. In addition, overexpression of *IlVP* enhanced the accumulation of Na^+^ in tissues. On the one hand, these differences might be ascribed to enhanced sequestration of Na^+^ into the vacuole and maintained the balance between K^+^ and Na^+^, and increased the osmotic regulation ability, because of overexpressing of H^+^-PPase^38–40^. On the other hand, potassium is required for plant growth, tropisms, cell expansion, enzyme activity, ion homeostasis and stomatal movements^41^. Our study showed that the K^+^ concentrations in transgenic tobacco were higher than in the WT plants under salt stress. The increased accumulation of potassium is likely to be overexpression of the H^+^-PPase resulted in the enhanced K^+^ uptake and the release of organic acids, which contribute to an increased rhizosphere acidification and to enhanced phosphorus uptake, and thus improved salt tolerance in transgenic plants^42^.

## Conclusions

*IlVP* was cloned from *I*. *lactea*. *IlVP* expression was observed mainly in shoots, and its transcript abundance changed with increasing salt concentration and duration of exposure.

Phenotypes, the leaf relative water content, relative plasma membrane permeability, and concentrations of Na^+^ and K^+^ in roots, stems, and leaves were measured in response to treatment with different NaCl concentrations for 7 days. The transgenic tobacco plants displayed enhanced tolerance to NaCl stress compared with the WT plants. These results suggest that overexpression of *IlVP* in tobacco plants enhances sequestration of Na^+^ into vacuoles to alleviate Na^+^ toxicity in the cytoplasm, further maintaining cellular K^+^ and Na^+^ homeostasis and cell membrane stability, thereby enhancing tobacco salt tolerance.

## Materials and Methods

### Material culture and main experimental reagents

Seeds of *I*. *lactea* Pall. var. *chinensis* (Fisch.) Koidz. were collected from the National Experiment Station of Precision Agriculture, Xiao Tang Shan, China, located approximately 55 km from Beijing (39°34′ N, 116°28′ E). Plump seeds were sterilised with sodium hypochlorite solution (5%) for 5 min, rinsed thoroughly with distilled water, incubated in 40 °C water for 56 h, and then germinated on moistened filter paper for 10 days at 25 °C in the dark. After plumule emergence, uniform seedlings were transferred to plastic containers (19 cm long, 13.5 cm wide and 7.5 cm high) containing modified Hoagland’s solution (2 mM KNO_3_, 1 mM NH_4_H_2_PO_4_, 0.5 mM Ca(NO_3_)_2_·4H_2_O, 0.5 mM MgSO_4_·7H_2_O, 60 µM Fe-citrate, 92 µM H_3_BO_3_, 18 µM MnCl_2_·4H_2_O, 1.6 µM ZnSO_4_·7H_2_O, 0.6 µM CuSO_4_·5H_2_O, and 0.7 µM (NH_4_)_6_-Mo_7_O_24_·4H_2_O) for 5 weeks. The nutrient solution was renewed every 3 days. All seedlings were grown in the same chamber under a day/night cycle of 16 h/8 h at 25 °C/18 °C, a relative humidity of 50%–60%, and 600 µmol m^−2^ s^−1^ photosynthetically active radiation.

### Cloning of *IlVP*

A pair of degenerate primers, P1 and P2, were designed based on *VP* gene sequences in GenBank. Six-week-old seedlings of *I*. *lactea* were treated with 100 mM NaCl for 24 h. After treatment, fresh roots (100 mg) were washed in sterile water and then ground in liquid nitrogen. Total RNA was extracted using a Takara RNA extraction kit. After synthesis of cDNA using a First-Strand PrimeScript RTase cDNA synthesis kit, reverse transcription and PCR amplification were conducted. The PCR protocol was as follows: 94 °C for 2 min, followed by 30 cycles of 94 °C for 30 s, 56 °C for 30 s, and 72 °C for 1 min, and a final step of 72 °C for 10 min. The amplified fragment was sequenced and analysed using the BLAST tool (http://www.ncbi.nlm.nih.gov/BLAST). *IlVP* 5′- and 3′-ends were obtained using Clontech SMARTer RACE and Takara 3′-Full RACE kits in accordance with the manufacturers’ instructions and the gene-specific primers P3 and P4, respectively (Supplementary Table 1). The ORF of *IlVP* was amplified using a Tks Gflex DNA Polymerase PCR kit with primers P5 and P6 (Supplementary Table 1).

### DNA sequence and phylogenetic analyses

The *IlVP* sequence was analysed, and the coding regions were predicted using DNAMAN 6.0 software. *IlVP* sequence homology analysis and phylogenetic tree construction was carried out using MEGA 6.0 software. The isoelectric point and molecular mass were predicted using the online Compute pI/Mw tool (http://web.expasy.org/compute_pi/).

### Expression analysis of *IlVP*

Six-week-old seedlings of *I*. *lactea* var. *chinensis* were treated with 200 mM NaCl for 24 h. After washing the roots in sterile water, 200 mg of fresh roots and shoots were ground separately in liquid nitrogen. Total RNA was extracted using a Takara RNA extraction kit, after which cDNA was synthesised using a PrimeScript RT Reagent Kit with gDNA Eraser. In addition, 6-week-old seedlings of *I*. *lactea* were treated with 0, 25, 50, 100, and 200 mM NaCl for 0, 6, 12, 24, and 48 h; total RNA was extracted, cDNA was synthesised as described above, and the expression of *IlVP* in shoots was analysed. Quantitative real-time RT-PCR (qRT-PCR) was performed using SYBR Premix Ex *Taq* II (Perfect Real Time) (Takara) on a StepOnePlus Real-Time PCR system (ABI) to monitor the amplification of each cDNA fragment. The qRT-PCR amplification was performed using the *IlVP-*specific primer pair P7 and P8. *Actin* amplified with the primer pair A1 and A2 was used as an internal reference in the qRT-PCR analysis. All experiments were carried out with three biological replicates.

The qRT-PCR protocol consisted of three steps: predenaturation at 95 °C for 30 s; PCR amplification for 40 cycles of 95 °C for 5 s and 60 °C for 1 min; and finally, dissociation, consisting of 95 °C for 10 s, 65 °C for 5 s, and 95 °C for 5 s.

### Construction of plant expression vectors

The plasmid containing the *IlVP* gene and the plasmid pBI121 were cut with QuickCut SmaI and QuickCut ScaI, respectively, and incubated for 15 min at 30 °C. The two fragments were ligated in accordance with the DNA Ligation Kit Version 2.1 manual. A plant expression vector that included the CaMV 35S promoter, a NOS terminator and the *IlVP* gene was constructed. The recombinant plasmid was transformed into *Escherichia coli* strain DH5α. The cells were screened for kanamycin and rifampicin resistance. After purification of cells harbouring the recombinant plasmid, restriction enzyme digestion was performed.

### Genetic transformation and identification of transgenic tobacco

Chemically competent cells of *Agrobacterium tumefaciens* strain EHA105 were prepared. After fusing the plant expression vector into EHA105 cells using the freeze–thaw method, tobacco strain ‘W38’ was transformed using the leaf-disc method. Tobacco leaf strips that showed expansion after placement on Murashige and Skoog (MS) culture medium lacking kanamycin were transfected by *Agrobacterium* for 7 min, blotted with sterile filter paper to remove excess liquid, and co-cultured for 2–3 days. The leaf strips were placed on kanamycin-containing differentiation medium (MS medium supplemented with 1 mg L^−1^ 6-benzylaminopurine, 0.1 mg L^−1^ naphthaleneacetic acid, 50 mg L^−1^ kanamycin, and 500 mg L^−1^ carbenicillin). Callus was visible after 4 weeks of culturing on this medium. When generated shoots were 1–3 cm tall, they were transplanted onto MS medium supplemented with 50 mg L^−1^ kanamycin and 500 mg L^−1^ carbenicillin. Disinfected leaf strips were used as a control and were cultured on MS medium. When space was insufficient for growth, the generated shoots were transplanted into plastic culture pots containing a mixture of vermiculite and perlite (v/v, 3:1) and grown under a 16 h/8 h (light/dark) photoperiod and a light intensity of 600 µmol m^−2^ s^−1^ at 25 °C and 60% relative humidity. The plants were watered every 2 days with Hoagland’s solution. Total genomic DNA was extracted from the leaves of regenerated and WT plants in accordance with the Takara MiniBEST Plant Genomic DNA Extraction kit manual. PCR amplification was carried out with a TksGflex DNA Polymerase kit following the manufacturer’s instructions using plasmid DNA as a positive control, WT plant DNA as a negative control, and the following pair of specifically designed primers: F1 (5′-CATTGCTGGGATGGGTTC-3′) and R1 (5′-TCGTGGCTGCTCCTGTTC-3′). The PCR protocol consisted of pre-denaturation at 94 °C for 1 min, followed by 30 cycles of denaturation at 98 °C for 30 s, annealing at 55 °C for 15 s, and elongation at 68 °C for 1 min, followed by storage at 4 °C.

### Southern Northern blot assays

Preparation of the Southern blot probe was carried out using a PCR DIG Probe Synthesis kit (Beijing Mylab Corporation). DNA samples (30 µg) were cut with DraI. The enzyme-digested product was purified and electrophoresed on a 1% agarose gel at 20 V. A capillary siphon was used for transfer of the purified product. After prehybridisation for 2 h, a radiolabeled probe was added and hybridised overnight.

DIG-PCR amplification was used to label probes for the Northern blot assay. After carrying out 1.1% formaldehyde denaturing agarose gel electrophoresis for 3 h at 50 V, a capillary siphon was used for transfer of the purified product. After prehybridisation for 2 h, the radiolabeled probe was added and hybridised overnight. Membrane washing and signal detection were conducted using a DIGD-210 Hybridization Detection II kit in accordance with the manufacturer’s instructions.

### Assessment of salt tolerance of transgenic tobacco

Transgenic tobacco (T4 and T18) and WT plants grown under identical growth conditions were irrigated for 7 days using Hoagland’s solution containing 0, 50, 100, or 200 mM NaCl. Seven biological replicates were conducted, with three seedlings of each strain used per replicate. Root, stem, and leaf fresh weights, leaf relative water content, relative electrical conductivity, and Na^+^ and K^+^ concentrations were measured. After fresh weight measurements, roots, stems, and leaves were oven-dried at 80 °C to a constant weight, and the dry weight of each organ was recorded. The leaf relative water content and relative electrical conductivity were measured using Gao’s method^43^. Na^+^ and K^+^ concentrations in roots, stems, and leaves were measured using a flame emission spectrophotometer.

### Tonoplast vesicles isolation and Western blot

According to the method of Wang *et al*. (2000)^44^ with minor modifications, tonoplast enriched membrane vesicles were isolated. Briefly, about 100 mg leaves of WT and transgenic tobacco (T4 and T18) were selected under 200 mM NaCl treatment for 10 days, which were homogenized in extraction medium (pH 7.8) containing 250 mM mannitol, 1 mM DTT, 3 mM EGTA, 1% (w/v) PVP, 0.25 mM PMSF, 100 mM Tricine, 3 mM MgSO_4_. The homogenate was filtered using four layers of cheesecloth that was centrifuged at 12,000 × g for 15 min at 4 °C. Subsequently, these supernatant were centrifuged at 300,000 × g for 45 min and was suspended in suspension buffer (pH 7.5) with 250 mM mannitol, 2 mM DTT, 3 mM EGTA, 10 mM Hepes. The microsomal membrane vesicle suspension was loaded on a 1%/18% (w/w) Dextran T_70_ gradient in suspension buffer and then centrifuged at 100,000 × g for 2 h. The tonoplast-enriched membrane vesicle fraction located at the 1%/8% (w/w) Dextran T_70_ interface was carefully collected, diluted 4–5 fold with dilution buffer (pH 7.0) with 1 mM DTT, 3 mM MgSO_4_, 50 mM Hepes, 0.2 mM PMSF and then centrifuged at 300,000 × g for 45 min. For western blot, 100 μg tonoplast proteins were separated using 12% SDS-PAGE (sodium dodecyl sulfate polyacrylamide gel electrophoresis), and was immunoblotted with antibody against H^+^-PPiase (Agrisera, Vännäs, SWEDEN). Blots were performed according to describe the methods of Sarafian *et al*. (1999)^45^ and Venancio *et al*. (2014)^46^.

### Statistical analysis

Excel 2010 was used for data processing. SAS11.0 was used for variance and clustering analyses.

## Electronic supplementary material

Supplementary information
