# Supplementary material for: Molecular cloning and functional characterisation of an H+-pyrophosphatase from Iris lactea
Source: Sci Rep. 2017 Dec 19;7:17779. doi: 10.1038/s41598-017-18032-3 (PMC5736642; doi:10.1038/s41598-017-18032-3)
Supplement: Supplementary file 1 — Supplementary information [file 41598_2017_18032_MOESM1_ESM.pdf]

Molecular cloning and functional characterization of an H^+-pyrophosphatase from *Iris lactea*

Authors: Lin Meng*, Shanshan Li, Jingya Guo, Qiang Guo, Peichun Mao, Xiaoxia Tian

Institutional addresses: Beijing Research and Development Centre for Grass and Environment, Beijing Academy of Agriculture and Forestry Sciences, Beijing 100097, P. R. China

***Corresponding author:** Lin Meng, E-mail: menglin9599@sina.com

Tel: +86-10-51503345

Fax: +86-10-51503297

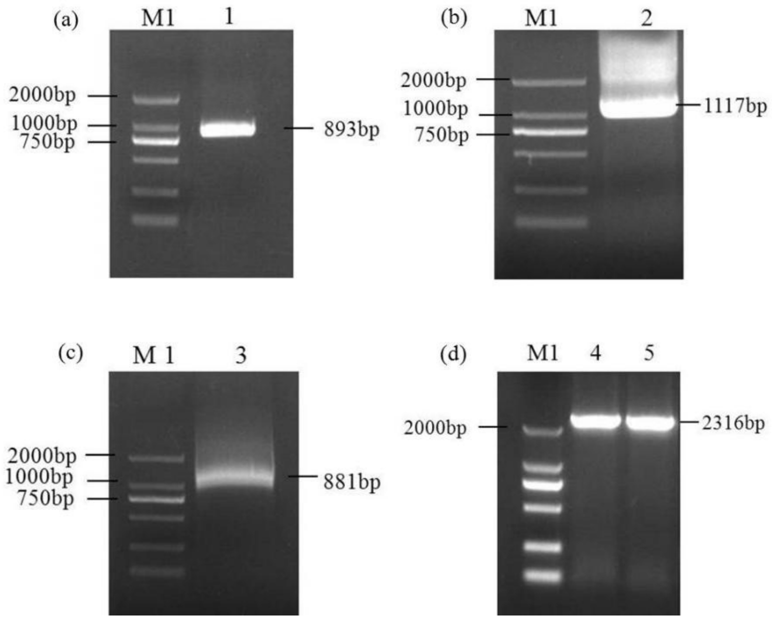

Supplementary Figure 1 Electrophoresis of *IlVP* cDNA clones. (a) 1: RT-PCR products of the *IlVP* fragment; (b) 2: 3′ RACE product of the *IlVP* gene; (c) 3: 5′ RACE product of the *IlVP* gene; (d) 4, 5: PCR product of the full-length *IlVP* gene.

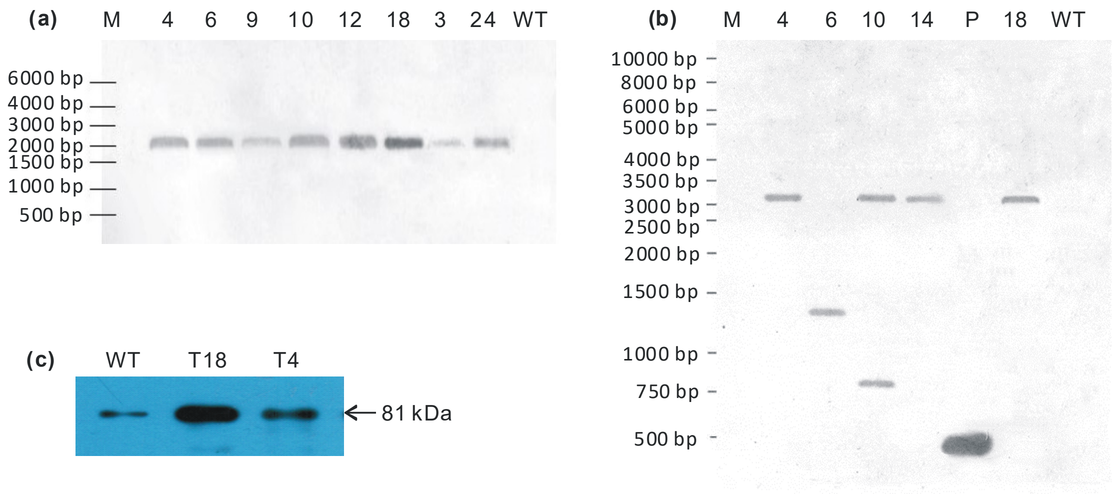

Supplementary Figure 2 **a**. Northern blot analysis of *IlVP*-transgenic tobacco plants. Lines 4, 6, 9, 10, 12, 18, 3, and 24 and WT correspond to individual transgenic tobacco lines and the wild type, respectively. **b**. Southern blot analysis of *IlVP*-transgenic tobacco plants. Lines P (positive control) 4, 6, 10, 14, 18 and WT correspond to individual transgenic tobacco lines and the wild type, respectively. **c.** Western blot analysis of IlVP protein in WT and transgenic tobacco lines (T4 and T18) under 200 mM NaCl treatment for 10 d, respectively.

Supplementary Table 1 Primer sequences used in the experiments

Primer	Sequence (5'-3')
P1	TATGGTGATGAYTGGGAAGG
P2	GCAATRCCWCCAGCATTRTCA
P3	CAACATCAGCAGCTTTAGTGTAGATA
P4	TATGGCCCCATCAGTGACAATGCT
P5	TCCCCCGGGATGGTGGCGGCGATGCT
P6	CGAGCTCTTAGAAGATCTTGAAGAGGATGCC
P7	CGAACTGACTGCTATGATGTACCC
P8	CCAACTAACAACCGCAATACCA
A1	TATTGTGCTGGATTCTGGTGATG
A2	GGAGGATAGCATGGGGAAGAG
